# Spatially Offset Raman Spectroscopy toward In Vivo Assessment of the Adipose Tissue in Cardiometabolic Pathologies

**DOI:** 10.1021/acs.analchem.4c01477

**Published:** 2024-06-12

**Authors:** Ewa Stanek, Zuzanna Majka, Krzysztof Czamara, Joanna Mazurkiewicz, Agnieszka Kaczor

**Affiliations:** † Doctoral School of Exact and Natural Sciences, Jagiellonian University, 11 Lojasiewicza Str., 30-348 Krakow, Poland; ‡ Jagiellonian Centre for Experimental Therapeutics (JCET), Jagiellonian University, 14 Bobrzynskiego Str., 30-348 Krakow, Poland; § Faculty of Chemistry, Jagiellonian University, 2 Gronostajowa Str., 30-387 Krakow, Poland

## Abstract

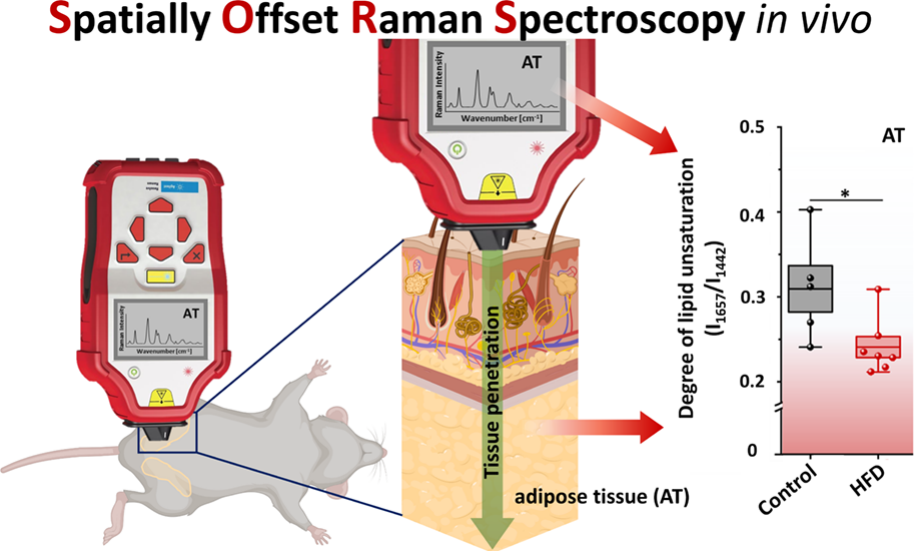

Spatially offset Raman spectroscopy (SORS) enhanced the capabilities of Raman spectroscopy for the depth-resolved analysis of biological and diffusely scattering samples. This technique offers selective probing of subsurface layers, providing molecular insights without invasive procedures. While SORS has found application in biomedical research, up to now, studies have focused mainly on the detection of mineralization of bones and tissues. Herein, for the first time, SORS is used to assess the soft, organic tissue beneath the skin’s surface. In this study, we demonstrate the diagnostic utility of a hand-held SORS device for evaluating the chemical composition of the adipose tissue. We compared perigonadal white adipose tissue (gWAT) in a murine model of atherosclerosis, heart failure, and high-fat diet (HFD) induced obesity. Our results reveal distinct chemical differences in gWAT between HFD-fed and control mice, showcasing the potential of SORS for intravital adipose tissue phenotype characterization. Furthermore, our findings underscore the effectiveness of SORS as a valuable tool for noninvasive assessment of the adipose tissue composition, holding potential diagnostic significance for metabolic disorders.

In the past few years, significant progress has been made in enhancing the capacity of Raman spectroscopy for depth-resolved analysis of biological tissues and other diffusely scattering samples.^1^ The introduction of spatially offset Raman spectroscopy (SORS)^2^ and the development of its variants^3,4^ enabled the selective probing of subsurface layers^5^ providing molecular information without the need for invasive procedures.^6^ The technique itself operates on the premise that offsetting the Raman signal collection zone from the point of laser illumination mitigates the influence of the outer layer, facilitating the analysis of structures below the surface.^7^ Thus, SORS has been successfully employed in various applications, from drug testing,^8,9^ quality control,^10^ and airport security^11^ to forensic science^12^ and diagnostics,^3^ making it a versatile tool across different industrial and scientific domains.

In the case of medical implementation, SORS also offers some advantages over traditional clinical imaging, contributing to further expanding transcutaneous measurements. Unlike magnetic resonance imaging (MRI), computed tomography (CT), and positron emission tomography (PET), it provides detailed characterization of the subsurface structures without contrast agents or radioactive tracers. Additionally, it surpasses ultrasound in its ability to penetrate opaque or turbid media. The first attempts to incorporate SORS into biomedical research^13^ began in 2006, when SORS spectra of bone were obtained, contributing to the development of *in vivo* bone disease detection in mice^14^ and humans.^15,16^ Further studies have focused on monitoring bone mineralization in tissue engineering^17^ and bone healing in rat calvarial defects.^18^ However, most importantly, SORS-based techniques have been used to characterize soft tissues, identifying microcalcifications in breast tissue phantoms,^19^ nonmelanoma cancer subtypes in skin biopsy samples,^20^ and skin changes caused by sunburn studied on human volunteers,^21^ thus having introduced entirely novel possibilities for a diverse array of analytical applications. Moreover, integrating SORS with surface-enhanced Raman spectroscopy (SESORS) enables signal detection at greater depths^22,23^ to reveal and target disease states by sensing glucose concentrations,^24,25^ neurochemicals,^26^ or the presence of tumor spheroids.^27^ However, incorporation of such methods involves introducing specific Raman nanotags^28^ into the body, which requires additional invasive procedures.

SORS is commonly utilized to study inorganic compounds and *in vivo* only through the skin, to diagnose hard tissue,^14,15^ which is dense and characterized by very intense Raman bands. Yet, inorganic matter such as hydroxyapatite and carbonates (components of bones) are much easier to identify even if present as minor deposits^29,30^ compared to soft tissue signals. However, SORS has not been applied transcutaneously to study pathologies involving soft tissues *in vivo*.

As the adipose tissue is a promising target for therapeutic interventions, there is an urgent need to address the simultaneous rise of obesity and cardiometabolic diseases^31^ and the development of dedicated research equipment. An example of such systems proved to be fiber probe-based Raman devices, increasingly used in clinical diagnoses.^32^ Up-to-date studies have indicated their benefits in the analysis of the adipose tissue phenotype, also intraoperatively on patients undergoing coronary bypass surgery^33^ as well as in determining lipid accumulation in the subcutaneous layer of the skin in anesthetized hamsters.^34^ Nevertheless, other than endoscopic fiber optic probes are usually designed for surface measurements and are not intended for deep-tissue penetration.

In the present work, we demonstrate the potential diagnostic application of a commercially available hand-held SORS in the Raman-based evaluation of the murine adipose tissue. In the murine study, we compared the chemical composition of perigonadal white adipose tissue (gWAT) in control mice and transgenic models of cardiovascular pathologies (atherosclerosis, heart failure) as well as high-fat-diet-induced (HFD) induced obesity. The intravital measurements of Raman spectra highlighted differences between mice fed with the HFD. The proof-of-concept results show that SORS in the proposed experimental conditions enables the characterization of the perigonadal adipose tissue *in vivo*.

## Experimental Section

### Animals

Experiments were conducted on sex- and disease-specific animal groups, as described in Table 1.

**Table 1:** Description of the Mice Subgroups Used in the Study

**model**	**mouse strain**^a^	**diet**	**age**^b^	**sex**^c^	***N***^d^
	^1^C57BL/6J	CHOW	25	F	10
atherosclerosis	^2^*Apoe^–^/^–^/Ldlr^–^/*^–^	CHOW	25	F	5
	^3^FVB	CHOW	30	F	5
heart failure	^3^Tgαq*44	CHOW	60	F	8
obesity	^1^C57BL/6J	AIN-93G	10	M	8
		AIN-93G+B-glucan	10	M	8
		AIN-93G+butyrate	10	M	8
		HFD	10	M	8
		HFD+β-glucan	10	M	8
		HFD+butyrate	10	M	8

a Mice origin: ^1^Medical University of Bialystok, Experimental Medicine Centre, Bialystok, Poland; ^2^Department of Human Nutrition, University of Agriculture, Krakow, Poland; ^3^Medical Research Centre of the Polish Academy of Sciences, Warsaw, Poland.

b Age in weeks.

c Sex: F, female; M, male.

d *N*, number of individuals.

The study used female mice with developed atherosclerosis^35^ (*Apoe*^*–/–*^*/Ldlr*^*–/–*^ model) and heart failure^36^ (Tgαq*44 model) with C57BL/6J and FVB as control groups, respectively. Male C57BL/6J mice (obesity model group) at the age of 6 weeks were fed one of the selected diets for 4 weeks. Diets were based on either control formula^37^ (AIN-93G, ZooLab) or HFD (60 kcal% of fat +1% of cholesterol, ZooLab) with no additional supplementation or enriched with 5% w/w sodium butyrate (Sigma-Aldrich) or 4% w/w diet supplement containing 80% pure β-glucan (1,3/1,6D) obtained from*Saccharomyces cerevisiae* (RawDietLine β-Glucan, Pokusa) giving in total 6 experimental groups (AIN-93G, AIN-93G+butyrate, AIN-93G+β-glucan, HFD, HFD+butyrate, HFD+β-glucan), each comprising 8 individuals. All tested animals had access to daily provided diets and water *ad libitum*. Described procedures involving animals were approved by the Local Animal Ethics Commission (Krakow, Poland, identification code: 26/2019) and conducted according to the Guidelines for Animal Care and Treatment of the European Communities and the Guide for the Care and Use of Laboratory Animals published by the US National Institutes of Health (NIH Publication No. 85–23, revised 1996).

### Resolve SORS

Measurements were carried out using spatially offset Raman spectroscopy (Resolve hand-held SORS, Agilent) equipped with an excitation laser line of 830 nm. All measurements were made using the nose cone with through-the-barrier mode. The spectral range of the SORS instrument was 350–2000 cm^–1^ with a 10 cm^–1^ spectral resolution. Raman spectra were acquired with a 0.2 s exposure time per spectrum. To verify at which settings the gWAT-derived spectra would be obtained, offsets from 0.0 to 5.5 mm were tested. For this purpose, skin and gWAT were extracted from a control C57BL/6J male mouse. Tests experiments were performed on three phantom samples: 1/the layer of skin (0.6 mm) and gWAT (4 mm) were stacked, 2/skin and gWAT were separated by the polypropylene plate (1 mm) (Avantor), and 3/skin and gWAT were separated by the polypropylene plate with gWAT labeled with β-carotene. For gWAT labeling, the perigonadal fat pad was incubated for 1 h with 5 mM β-carotene (Merck, 1065480) dissolved in inhibitor-free tetrahydrofuran (THF, Sigma-Aldrich, 401757). *Post mortem* measurements of atherosclerosis, heart failure, and obesity (HFD, 4-week diet) model groups were taken after the lethal dose of a mixture containing ketamine and xylazine (100 mg of ketamine/10 mg of xylazine per kilogram of body weight) was administered by intraperitoneal injection. Spectra were collected from the area where the testicles/ovaries and gWAT are located. The measurement site was shaved and disinfected with 70% ethanol each time. At least three measurements were collected from each mouse using the maximum laser power, ca. 430 mW. To prevent possible tissue overburn in the HFD model (as mice skin at 10 weeks is considerably thinner), the lower laser power (ca. 313 mW) was used. *In vivo* experiments were performed on obesity model groups after diets with the selected dietary formulas were introduced for 2 weeks. To carry out measurements, mice were anesthetized with isoflurane (Aerrane, Baxter Sp. z o. o., 1.5 vol %) in an oxygen and air (1:2) mixture. The measurement site and procedures were the same as for the *post mortem* tests; however, spectra were collected using lower laser power (ca. 313 mW) to limit laser exposure as a safety precaution. After the procedure, mice were awakened and lived for another 2 weeks until they were sacrificed for another study.^38^ For *post mortem* and *in vivo* experiments, the chosen offset value was 5.5 mm.

### Raman Microscopy

Additionally, tissue measurements were performed with the confocal Raman microscope (WITec alpha300, Ulm, Germany) equipped with a 532 nm laser, a UHTS 300 spectrograph (600 grooves mm^–1^ grating), and a CCD detector (DU401A-BV-352, Andor, UK). Raman spectra were collected with a 1 μm sampling density in the *z-*axis with a 1 s exposure time and 10 accumulations per spectrum using low laser power (ca. 2 mW) to avoid tissue overheating. The laser power was the maximal possible to use without overburn in the conditions of the experiment (the 532 nm laser line and high optical density arising from confocality of the incident radiation). The phantom model was measured with a 20× air objective (NA = 0.45, Nikon CFI S Plan Fluor ELWD, Japan) on a CaF_2_ glass slide.

### Data Analysis

Generated files acquired from the Resolve spectrometer were analyzed in the Command program. To do so, Agilent Offset and SORS data were extracted, that is, spectra collected from the inside of the tissue (including the outer layer) and spectra without barrier contribution (calculated using the appropriate algorithm), respectively. Then, the spectra of SORS and offset baselined automatically by the Resolve software were averaged per group and analyzed using the OPUS 7.2 program. The integral intensities of the bands at 1747, 1657, 1442, 809, 398 cm^–1^ were calculated in the 1781 to 1723, 1682 to 1631, 1488 to 1401, 826 to 781, 420 to 378 cm^–1^ spectral ranges, respectively. The ratio of the bands at 1657/1442 cm^–1^ was used to determine the degree of lipid unsaturation. All data were compared in the Origin Pro 9.1 program using the two-way ANOVA variance analysis with the Scheffé *post hoc* test and the Student's *t* test for independent samples to characterize the differences in the chemical distribution in all pairwise comparisons for each of the studied groups. If the *p* parameter was less than 0.05, then, differences were identified as statistically significant. The normality of the distribution of each data set was checked by the Shapiro-Wilk test (*p* > 0.05) and the equality of variances (*p* > 0.05) by the Levene's test. Preprocessing of the spectra obtained by Raman microscopy, that is, the baseline correction using automatic polynomial regression of degree 3, was performed via the WITec Project Plus 5.1. software. All spectra were normalized using vector normalization in the 1800–600 cm^–1^ range with the OPUS 7.2 program.

## Results and Discussion

### Phantom Samples: Validation of the Adipose Tissue In-depth Measurements

**Figure 1:**
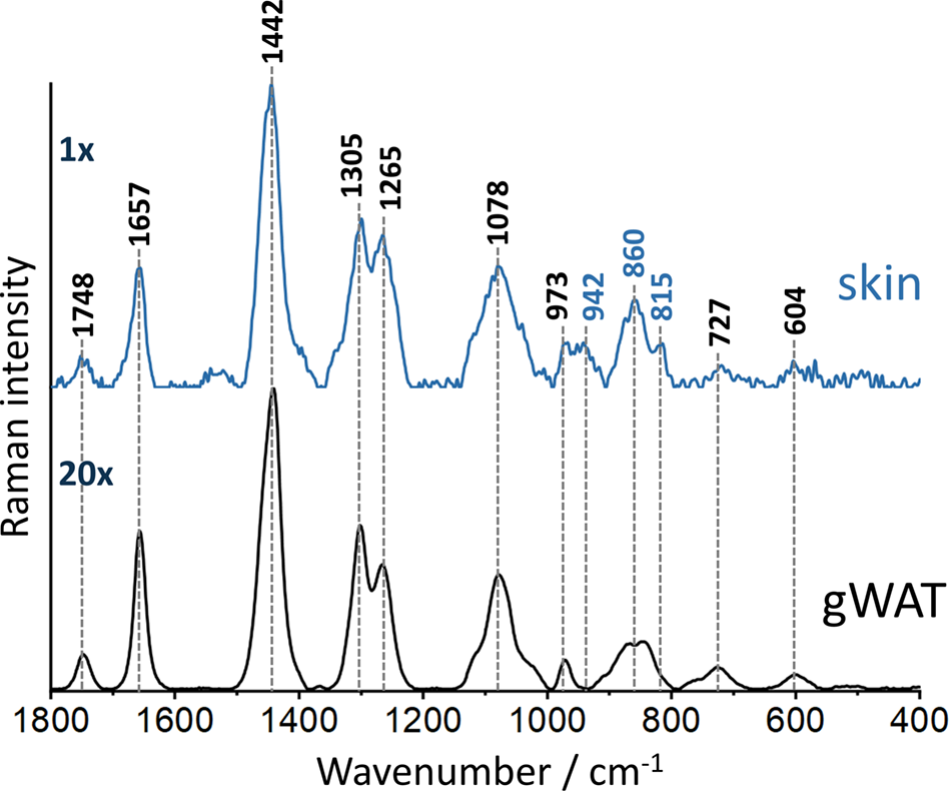
Representative SORS spectra collected from skin and gWAT. Spectra were acquired at a 0.0 offset and presented as maximally extended in the *y*-axis. X is the factor that enables comparing intensities of spectra, 20× means that the spectrum is 20 times more intense than the spectrum denoted as 1×.

This study aimed to prove whether commercially available equipment based on SORS methodology is effective for the adipose tissue examination *in vivo via* the layers of skin and the hypodermal region. In cardiometabolic diseases, visceral white adipose tissue is highly prone to alterations;^39^ hence, gWAT, as the most abundant adipose tissue depot,^40^ was selected. The hypodermal region is also a layer of the white adipose tissue^41^ which is spectrally similar to gWAT that complicates the measurements. Therefore, as a first step, prior to measurements *in vivo*, SORS profiling was performed on various *ex vivo* tissue phantoms. To mimic the anatomy (Figure S1, Supporting Information) of the measurement site, the first phantom consisted of a layer of skin and peritoneum, which were positioned directly on the gWAT. SORS spectra of both of these layers were recorded (Figure 1) to distinguish them in spectra obtained in further experiments. Bands at 1748, 1657, 1442, 1305, 1265, 1078, and 973 cm^–1^ represent vibrations linked to lipid unsaturation and hydrocarbon chains^42,43^ associated with the adipose tissue. Additionally, collagen signals are visible at 942, 860, and 815 cm^–1^ representing ν(C–C) backbone vibrations and 727 cm^–1^ corresponding to the C–C stretching mode of the proline ring.^35,44^

**Figure 2:**
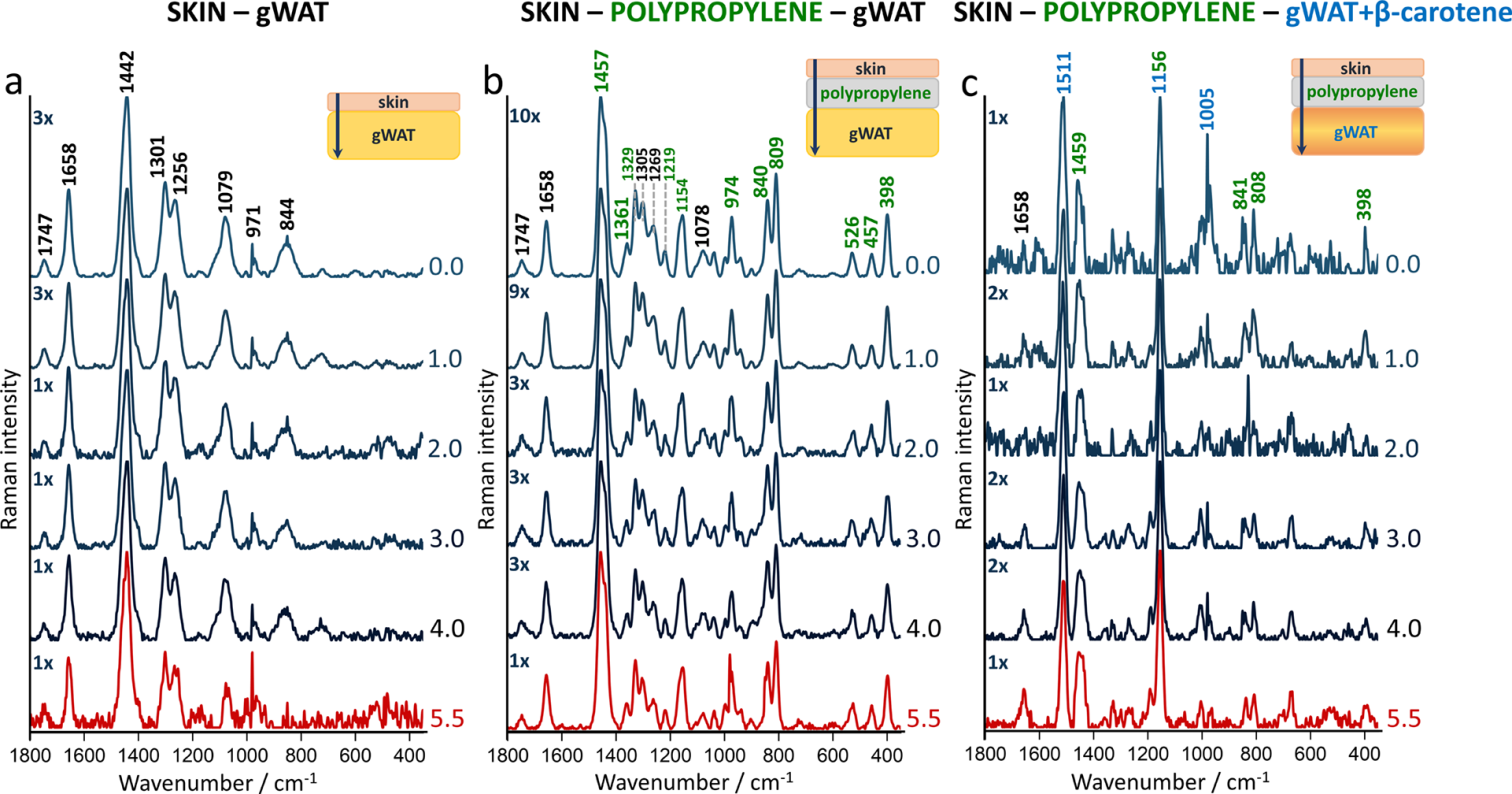
Spatial offset measurements confirm Raman signals from perigonadal white adipose tissue. Raman spectra collected from different offsets (0 to 5.5) where (a) layer of skin and gWAT were stacked (b) separated by the polypropylene plate and (c) separated by the polypropylene plate with gWAT labeled with β-carotene. All spectra were normalized to the highest band in the red spectrum. X is the factor that enables comparing intensities of spectra, 3× means that the spectrum is three times more intense than the spectrum denoted as 1×, etc.

In SORS measurements, a range of offsets from 0 to 5.5 changed every 0.5 mm (Figure S2, selected offsets in Figure 2) and were used to acquire spectra at slightly different depths. Interestingly, in the skin-gWAT phantom, from the starting point of the 0.0 offset, a standard lipid-specific profile is visible (Figure 2a). Regardless of the chosen offset, the obtained SORS spectra feature some lipid bands, however differing in the total and relative intensity, as indicated next to the *y*-axis (Figure S2a). In particular, the intensity of the 1747 cm^–1^ band attributed to triacylglycerols increases with the offset increase (Table S1a).

To confirm that the acquired Raman spectra originated predominantly from gWAT and not the skin hypodermis, a polypropylene plate (1 mm thick) was added between (Figure 2b).

Polypropylene provides very intense Raman signals^45^ (denoted in green), that is, 1361 cm^–1^ (CH_3_ wagging), 1154, and 809 cm^–1^ (C–C stretching) and bands at 974 and 840 cm^–1^ (CH_3_ rocking) that do not overlap with the keylipid bands including the signals at 1747 and 1658 cm^–1^, ester-originated C=O stretching vibrations and C=C stretching modes,^46^ respectively. The significant overlap is mostly seen at the 1457 cm^–1^ band corresponding to the CH_2_ bending vibration that coincides with the signal at 1442 cm^–1^ (the CH_2_ scissoring vibrations). Therefore, the intensity changes in the bands associated with the adipose tissue and plastic could be tracked, which is particularly pronounced at a 5.5 mm spectral change. SORS provides high-quality spectra with bands originating from polypropylene and lipids already visible at the lowest measured offset (Figure S2b). This can indicate that the Raman collection voxel extends several millimeters deep from the skin surface. Nonetheless, these measurements do not confirm the origin of the lipid signal. Therefore, an additional adipose tissue marker has been added to the skin-polypropylene-gWAT model. In this phantom, gWAT is labeled with β-carotene (Figure 2c), a carotenoid with a high Raman scattering cross-section at 830 nm due to pre-resonance, with characteristic bands^47^ (denoted in blue) at 1511, 1156, and 1005 cm^–1^. The carotenoid bands are evident in the spectra with all offsets (Figure S2c), which validates that only the signal associated with the significant depth is visible in the spectra from the 0.0 offset in every tested phantom. Considering the significant resonance band contributions from carotenoids in the barrier spectrum, the SORS spectrum (predominantly of the skin) is diminished by the barrier influence. Moreover, as the offset increases, the increase in the intensity of the 1658 cm^–1^ is seen alongside a decrease in the polypropylene band at 809 and 398 cm^–1^ (Table S1b). Overall, phantom models confirmed that in the chosen settings, the offset correlates with measured depths.

To demonstrate the benefits of the SORS technique over traditional Raman spectroscopy, for the phantom sample of skin-polypropylene-gWAT, measurements were made using a confocal microscope (Figure S3), where the spectra were collected horizontally going every 50 μm down in the *z*-axis. As expected using the confocal microscope, the marker bands from polypropylene are not observed and the spectra recorded at 0–350 μm predominantly show the band at 1450 cm^–1^ (the CH_2_ deformation vibrations) assigned to both protein and lipid content.^43^

### Transcutaneous Measurements Post Mortem

**Figure 3:**
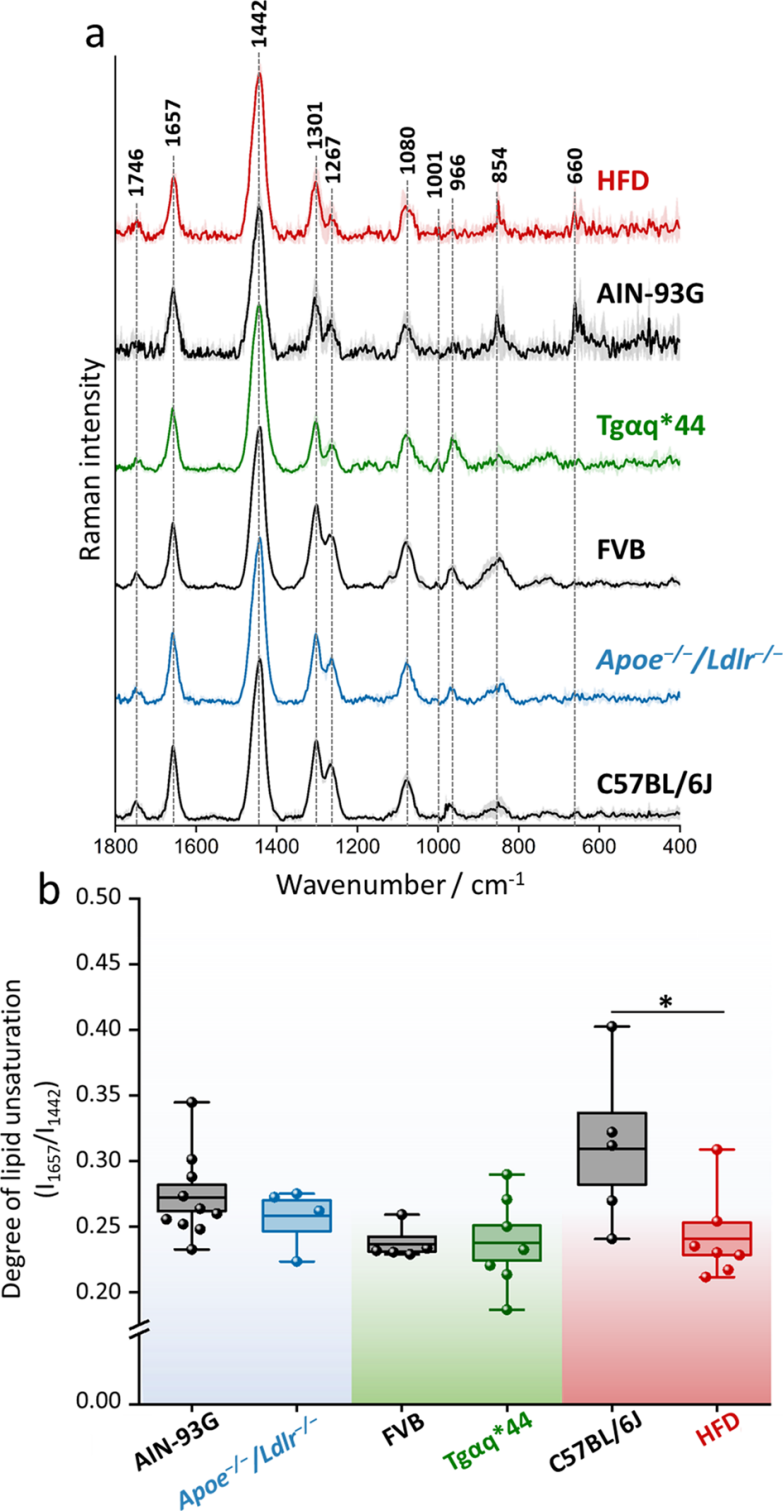
Chemical composition of the perigonadal white adipose tissue in mice with cardiometabolic diseases. Averaged Raman spectra with the standard deviation on each data point (a) of gWAT acquired *post mortem* from C57BL/6J, *Apoe*^*–/–*^*/Ldlr*^*–/–*^, FVB, Tgαq*44, and mice fed with a HFD for 4 weeks. The degree of lipid unsaturation (*I*_1657_/*I*_1442_) was calculated (b) for each studied group. Values shown in box plots: mean (horizontal line), SEM (box), minimal, and maximal values (whiskers). Statistical significance * *p* < 0.05.

For all subsequent mice measurements, an offset of 5.5 was used, as it confirmed the highest content of the signal derived from the gWAT. To validate described tissue phantoms, we analyzed gWAT transcutaneously in the murine models of cardiometabolic diseases (*Apoe*^*–/–*^*/Ldlr*^*–/–*^, Tgαq*44, HFD-induced obesity, respective controls: C57BL/6J, FVB, AIN-93G) and obtained SORS spectra, as shown in Figure 3a. All recorded spectra for each study group presented a typical triacylglycerol spectral profile. Yet, three additional bands have also emerged at 966, 854, and 660 cm^–1^ corresponding to lipids, tyrosine ring breathing mode, and collagen,^44^ respectively. Additionally, a comparative analysis of the spectra reveals that only the HFD shows a decrease in the lipid unsaturation degree, as determined by the ratio of n(C=C)/n(CH_2_) of the respective bands at 1657/1442 cm^–1^. The used parameter (as a ratio) is independent of the possible changes of the Raman intensity due to the potential temperature increase caused by the laser exposure. Ratiometric quantification (Figure 3b) reveals that the lipid unsaturation ranges between 0.2 to 0.4 for all studied groups. Statistical analysis shows a significant decrease in the degree of lipid unsaturation in the HFD compared to the AIN-93G. Similar changes were observed for the epididymal WAT isolated from the same animals^38^ and in our previous study showcasing the role of the diet on the adipose tissue chemical composition^48^ where the increase in the ratio of fat to protein and carbohydrates in the diet led to the accumulation of saturated fatty acids. The presented SORS data, obtained from *post mortem* and from isolated tissues, additionally confirm the accessibility to the gWAT and demonstrate the applicability of commercial hand-held SORS to the adipose tissue analysis.

### Adipose Tissue Screening In Vivo

**Figure 4:**
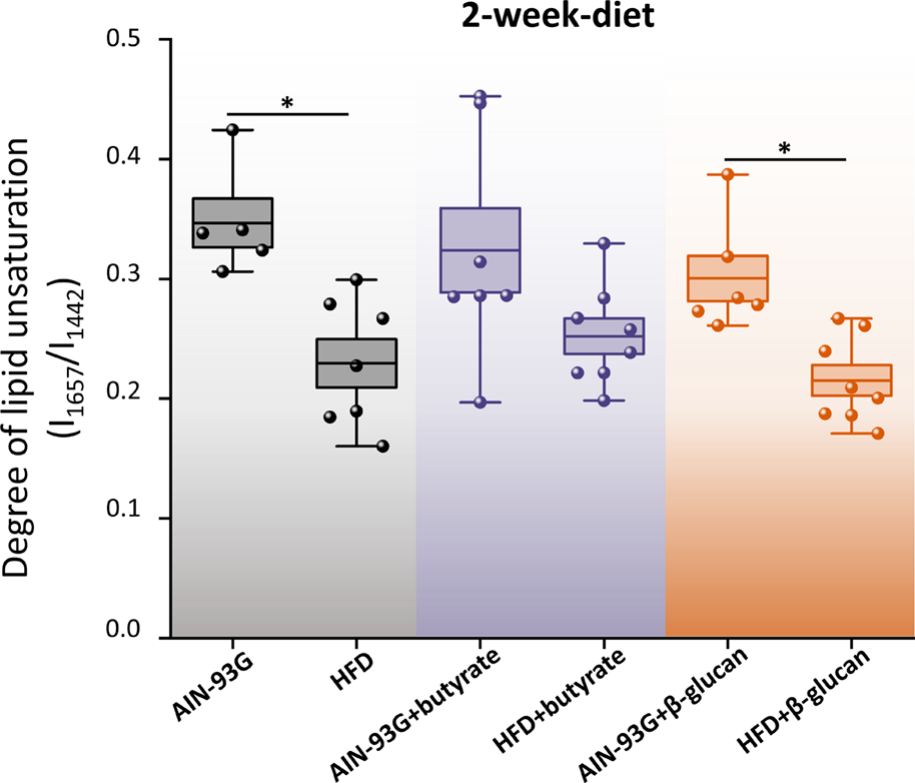
Diet-dependent changes in the lipid profile of perigonadal white adipose tissue. The degree of lipid unsaturation (*I*_1657_/*I*_1442_) was calculated for each group after 2-week exposure to the AIN-93G and HFD diet with or without additional supplements. Values shown in box plots: mean (horizontal line), SEM (box), minimal, and maximal values (whiskers). Statistical significance * *p* < 0.05.

Our primary goal was to employ SORS intravitally by eliminating the need to sacrifice mice. Transitioning to further research, we explored the 2-week dietary impact of HFD on adipose tissue *in vivo*. Moreover, to verify whether diet supplementation changes the lipid profile of gWAT, mice were fed with sodium butyrate, the sodium salt of the primary product of bacterial fermentation of unabsorbed carbohydrates,^49^ and β-glucan, a soluble dietary fiber.^50^ SORS measurements confirm that gWAT exhibits a decrease in the content of unsaturated lipids after just 2 weeks from the HFD introduction (Figure 4), statistically significant for HFD with no additives and β-glucan supplementation. Both analyzed additives are directly linked with anti-obesity properties,^50,51^ and we have demonstrated that they exert anti-obesity effect, however not related to the considerable chemical changes in the gWAT depot.^38^ As the same animals fed on HFD for 4 weeks were studied with gWAT measured *ex vivo* by fiber-optic Raman spectroscopy,^38^ we have a direct reference for our SORS results. Overall, both experiments exhibit a consistent trend concerning the selected diets, and the influence observed in our *in vivo* experiment became more pronounced as the duration of the supplementation extended (Figure S4). Our study validates the diagnostic potential of hand-held SORS in the analysis of the adipose tissue.

### Perspectives and Limitations

Although the SORS Resolve tool is not specifically designed for animal studies, we were able to obtain high-quality spectra of the adipose tissue from the perigonadal region and gather information on the progressive changes in the lipid profile induced by the HFD diet. It should be noted that using equipment nondedicated for clinical trials carries a risk of bias. However, the results gathered through the Resolve SORS provide a promising benchmark for studying the adipose tissue in mice and humans^39^*in vivo*. Nevertheless, there are certain limitations to be considered, particularly in the context of live animal research. A critical aspect is the selection of the laser power and the measurement site, which should be shaved prior to the procedure. During the *post mortem* experiments, it was observed that tissue and hair overburn can be induced by the high laser power; therefore, we used a short laser exposure time to minimize the temperature effect. Additionally, the laser power for intravital measurements was reduced to 313 mW. The maximum permissible exposure (MPE = 13,385.98 J/m^2^) and the actual laser exposure (19,936.30 J/m^2^ for a power of 313 mW) were calculated. Although the experimental exposure exceeds the MPE, no tissue damage was observed at 313 mW. However, considering the MPE limit, it would be advisable to use a lower laser power of ca. 156 mW or less (adjustable in Resolve as 33% laser power) in future experiments (beyond this proof-of-concept work). For a laser power of ca. 156 mW (or less) and other parameters unchanged, the actual laser exposure is within MPE limits (i.e., 9984.08 J/m^2^). It is also worth taking into account that the approach proposed in the paper is only appropriate if there is an explicit increase or decrease in the adipose tissue mass; therefore, when conducting dietary studies, it is crucial to plan the feeding period. Another challenge lies in the inability to continuously monitor alterations in the adipose tissue due to the gradual accumulation of body fat over time. Consequently, a methodical and rational approach is crucial to addressing these issues effectively. Moreover, it should be noted that the Resolve software baselines SORS spectra itself, limiting total control over the received data. Last but not least, sample heterogeneity and strong fluorescence background can hinder the efficacy of SORS measurements, limiting its depth resolution and sensitivity in some of the applications. Addressing these challenges demands continued advancements in instrumentation and data analysis techniques are required to fully harness the potential of SORS for measurements of various types of tissues *in vivo*.

For example, recent works on optimizing instruments and data analysis for diffuse Raman spectroscopy using computer modeling demonstrate significant improvements in performance over the conventional SORS.^52,53^

## Conclusions

SORS is a unique technique enabling measurements up to several mm in-depth depending on the type of the sample.^7^ Previously, SORS was used successfully in various models of diseases (phantom and *ex vivo* samples^14,18^ and *in vivo* for the analysis of hard tissues^13,54^) showing perspectives in diagnostics of osteogenesis imperfecta.^55^

In our study, we demonstrate that SORS can be a method of choice to study *in vivo* the chemical composition of perigonadal white adipose tissue (gWAT) via the layers of skin and peritoneum. gWAT is a soft tissue and as such a relatively weak scatterer compared to inorganic matter and also is chemically similar to the layer of the subcutaneous adipose. In the phantom experiments using layers of a good scatterer (polypropylene) and the carotenoid-labeled adipose tissue, we have demonstrated that the recorded SORS spectra contain information about gWAT with gWAT signals increasing in intensity with the increased offset.

The adipose tissue was chosen, as it is currently recognized as a promising and unexplored target for therapeutic interventions in cardiometabolic diseases. Hence, second, we used SORS in transcutaneous measurements *post mortem* to evaluate chemical changes of the adipose tissue in three cardiometabolic pathologies, that is, atherosclerosis (*Apoe*^*–/–*^*/Ldlr*^*–/–*^ model), heart failure (Tgαq*44 model), and obesity induced by high-fat diet (HFD). As gWAT is white, that is, the most lipid-accumulating type of the adipose tissue, significant chemical changes (a decrease in the lipid unsaturation ratio) occurred in this tissue due to obesity development.

The experiments in the obesity model were continued *in vivo* with and without antiobesity supplements, confirming further the applicability of SORS for evaluating the chemical composition of gWAT. We have shown using *in vivo* SORS that chemical changes induced in gWAT by two-weeks HFD intensified after another 2 weeks of feeding HFD the same animals, which was verified *post mortem* with the reference methods (fiber-optic Raman spectroscopy).^38^

Overall, our work demonstrates that SORS is an efficient analytical tool to study transcutaneously the adipose tissue *in vivo*, which has potential diagnostic applications in cardiometabolic pathologies.

## Supporting Information Available

Scheme of the studied site measured by SORS; SORS spectra of gWAT; Raman spectra of the phantom measured by the conventional Raman microscope; diet-dependent changes in the lipid profile of gWAT; and calculations of the integral intensities for phantom model (PDF)
