# Supplementary material for: Spatially Offset Raman Spectroscopy toward In Vivo Assessment of the Adipose Tissue in Cardiometabolic Pathologies
Source: Anal Chem. 2024 Jun 12;96(25):10373–9. doi: 10.1021/acs.analchem.4c01477 (PMC11209658; doi:10.1021/acs.analchem.4c01477)
Supplement: Supplementary file 1 — ac4c01477_si_001.pdf [file ac4c01477_si_001.pdf]

Supporting Information

Spatially Offset Raman Spectroscopy toward *in vivo* assessment of the adipose tissue in cardiometabolic pathologies

Ewa Stanek,[†,‡] Zuzanna Majka,[‡,§] Krzysztof Czamara,[‡] Joanna Mazurkiewicz,[†,§] and Agnieszka Kaczor[*,§]

[†]*Jagiellonian University, Doctoral School of Exact and Natural Sciences, 11 Lojasiewicza Str., 30-348 Krakow, Poland.*
[‡]*Jagiellonian University, Jagiellonian Centre for Experimental Therapeutics (JCET), 14 Bobrzynskiego Str., 30-348 Krakow, Poland.*
[§]*Jagiellonian University, Faculty of Chemistry, 2 Gronostajowa Str., 30-387 Krakow, Poland.*
[*]*corresponding author e-mail: agnieszka.kaczor@uj.edu.pl*

Content

<u>Figures</u>

Figure S1. Scheme of the studied site measured by SORS indicating the tissue layers used in *post mortem* and *in vivo* measurements.

Figure S2. SORS spectra confirm Raman signals originating from the perigonadal white adipose tissue.

Figure S3. Raman spectra of the skin-polypropylene-gWAT phantom measured by the conventional Raman microscope.

Figure S4. Comparison of the analysis of diet-dependent changes in the lipid profile of the perigonadal white adipose tissue carried out on spectra collected using SORS and fiber-optic Raman spectroscopy.

<u>Tables</u>

Table S1. Calculations of the integral intensities for phantom models used in the experiment.

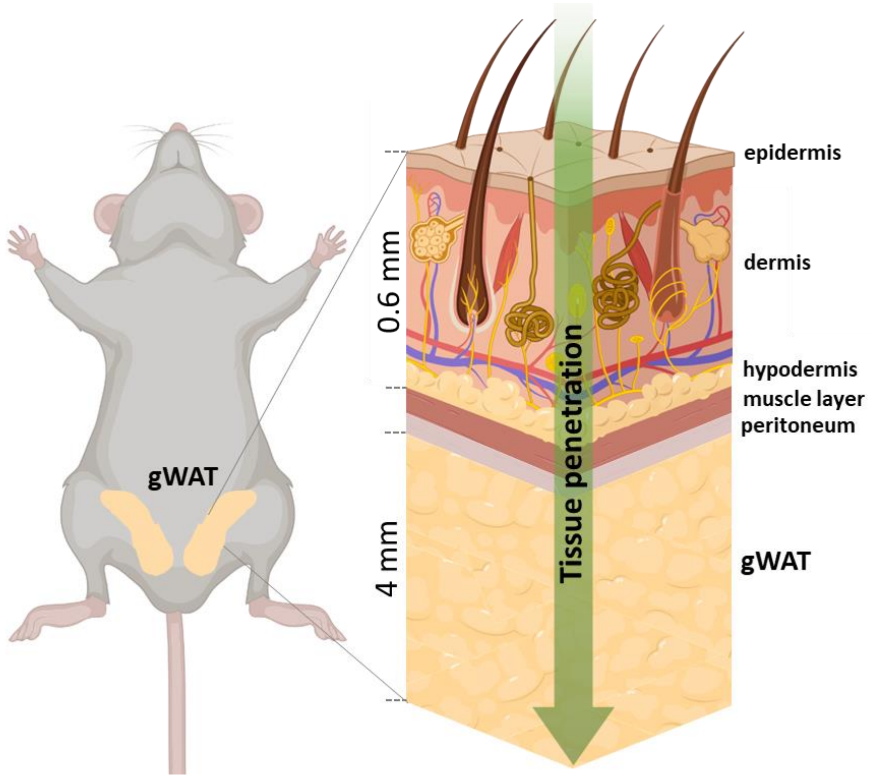

Figure S1. Scheme of the studied site measured by SORS indicating the tissue layers used in *post mortem* and *in vivo* measurements.

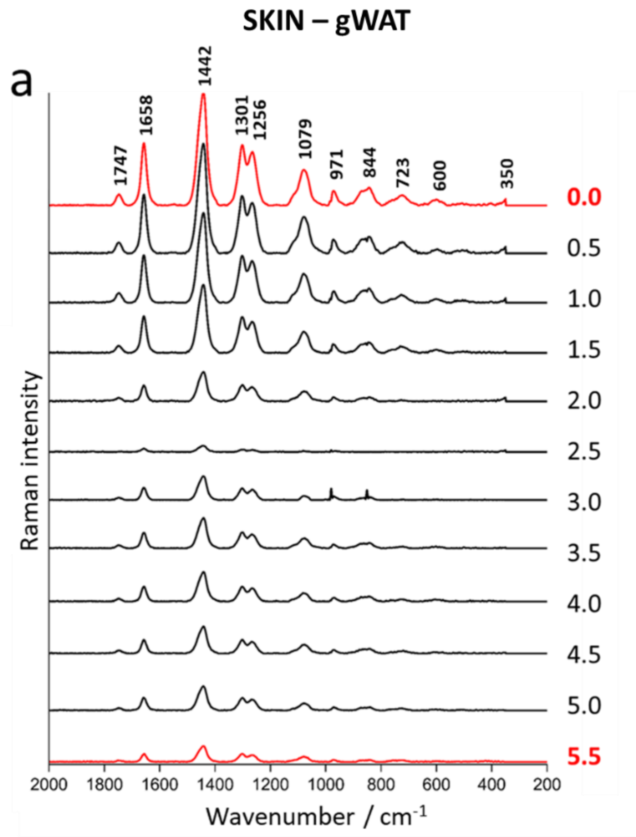

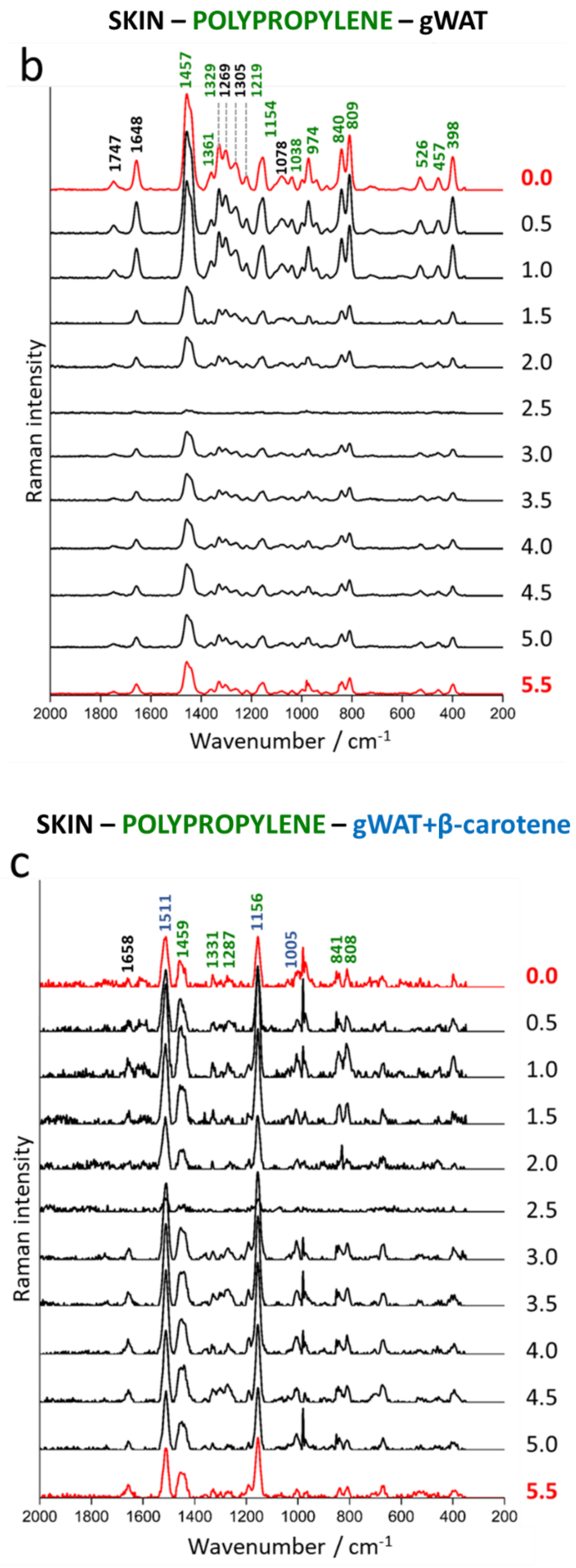

Figure S2. SORS spectra confirm Raman signals originating from the perigonadal white adipose tissue. Raman spectra collected from different offsets (from 0.0 to 5.5) where (a) layer of skin and gWAT were stacked, (b) separated by the polypropylene plate and (c) separated by the polypropylene plate with gWAT labeled with β-carotene.

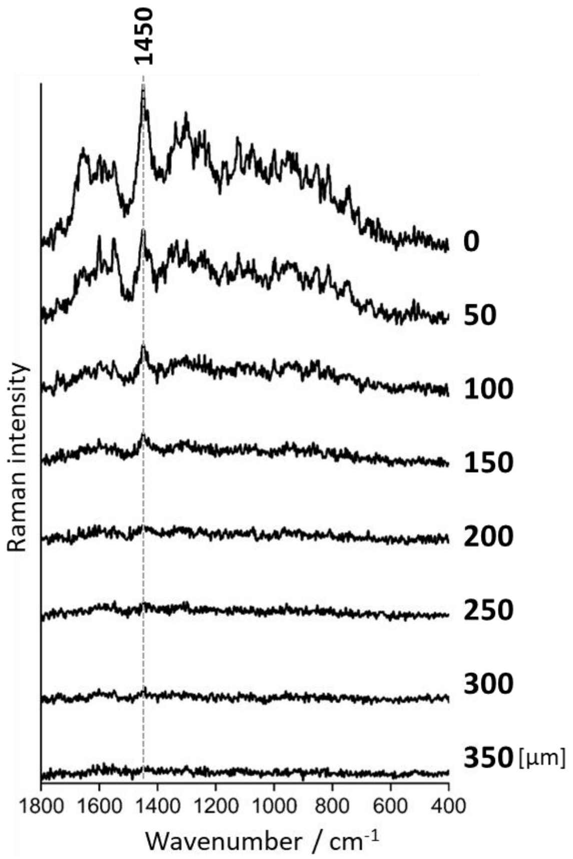

Figure S3. Raman spectra of the skin-polypropylene-gWAT phantom measured by the conventional Raman microscope. Spectra were collected horizontally every 50 μm lower in the *z*-axis from 0 (epidermis) to 350 μm.

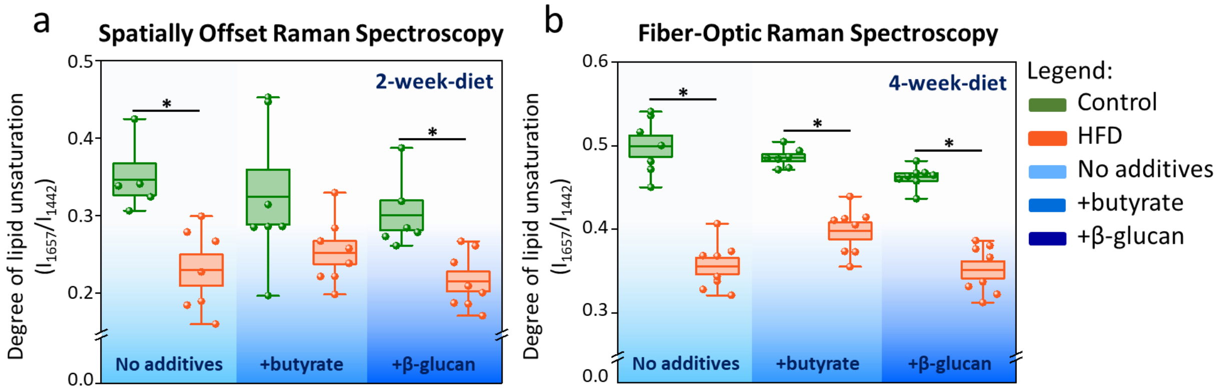

Figure S4. Comparison of the analysis of diet-dependent changes in the lipid profile of the perigonadal white adipose tissue carried out on spectra collected using SORS and fiber-optic Raman spectroscopy. The degree of lipid unsaturation (I_{1657}/I_{1442}) was calculated for each group after 2- and 4-week exposure to AIN-93G and HFD diet with or without additional supplements. Values shown in box plots: mean (horizontal line), SEM (box), minimal and maximal values (whiskers). Statistical significance * $p < 0.05$.

Table S1. Calculations of the integral intensities for phantom models used in the experiment. The ratio of the bands at 1657/1442 cm^{-1} and the integral intensity of 1747 cm^{-1} was used to determine the degree of lipid unsaturation and the level of triacylglycerols, respectively in the skin-gWAT model (a). For the skin-polypropylene-gWAT model, the ratios of the polypropylene bands (809 and 398 cm^{-1}) and unsaturated fatty acids (1658 cm^{-1}) were calculated (b).

a **SKIN – gWAT**

offset	1658/1442	1747
0	0,31524271	0,398845
0.5	0,30466017	0,449749
1	0,3033213	0,498667
1.5	0,30523006	0,465912
2	0,31897414	0,438846
2.5	0,36580775	0,675867
3	0,29240055	0,371312
3.5	0,32418668	0,629264
4	0,29704447	0,41137
4.5	0,32459833	0,369782
5	0,28797993	0,416803
5.5	0,2308117	0,608865

b **SKIN – POLYPROPYLENE – gWAT**

offset	809/1658	398/1658
0	1,42203	0,970734
0.5	1,442501	0,988667
1	1,36146	0,934966
1.5	1,125908	0,768474
2	1,217579	0,748524
2.5	0,350992	0,139554
3	1,319542	0,838319
3.5	1,075612	0,801072
4	1,320031	0,849423
4.5	1,176523	0,825432
5	1,255267	0,821724
5.5	1,215012	0,816911
